# Key proteins of invadopodia are overexpressed in oral squamous cell carcinoma suggesting an important role of MT1-MMP in the tumoral progression

**DOI:** 10.1186/s13000-021-01090-7

**Published:** 2021-04-20

**Authors:** Geovanni Pereira Mitre, Karolyny Martins Balbinot, André Luis Ribeiro Ribeiro, Maria Sueli da Silva Kataoka, Sérgio de Melo Alves Júnior, João de Jesus Viana Pinheiro

**Affiliations:** Laboratory of Histopathology and Immunohistochemistry, School of Dentistry, Cell Culture Laboratory, Federal University of Pará, Rua Augusto Corrêa, 01, Guamá, PA 66075110 Belém, Brazil

**Keywords:** Oral squamous cell carcinoma, Immunohistochemistry, Invadopodia, Neoplasm Invasiveness, Pathology

## Abstract

**Background:**

Oral squamous cell carcinoma (OSCC) is the most relevant malignant neoplasm among all head and neck tumours due to its high prevalence and unfavourable prognosis. Tumour invasion and metastasis that affect prognosis are result of a set of complex events that cells with invasive potential use to spread to other regions. These cells use several mechanisms to invade tissues, including a type of finger-like membrane protrusion called invadopodia. This study aims to investigate the immunoexpression of invaopodia related-proteins TKs5, cortactin, TKs4 and MT1-MMP in OSCC and correlate it to clinicopathological data.

**Methods:**

An immunohistochemical evaluation of fifty cases of OSCCs and 20 cases of oral mucosa (OM) were assessed. The expression of invadopodia proteins were analysed in comparison to normal tissue (OM) and correlated to different clinical-stage and histological grade of OSCC.

**Results:**

TKs5, cortactin, TKs4 and MT1-MMP were significantly overexpressed in OSCC when compared to OM (*p* < 0.0001). Among tumour stages, TKs5 showed a statistical difference in immunolabelling between stage I and III (*p* = 0.026). Cortactin immunolabelling was statistically higher in grade I than in grade II and III. No differences were seen on TKs4 expression based on tumour staging or grading. MT1-MMP was higher expressed and showed statistical difference between stages I and III and grades I compared to II and III.

**Conclusions:**

The invadopodia related-proteins were found to be overexpressed in OSCC when compared to OM, suggesting invadopodia formation and activity. Besides overexpressed in OSCC, cortactin, TKs4 and TKs5 showed no or ambiguous differences in protein expression when compared among clinical-stages or histological grades groups. Conversely, the expression of MT1-MMP increased in advanced stages and less differentiated tumours, suggesting MT1-MMP expression as a promising prognostic marker in OSCC.

## Background

Oral squamous cell carcinoma (OSCC) is the most relevant tumour in the head and neck region to its high prevalence and aggressiveness [1, 2]. Despite all developments in cancer treatment, OSCC still have a poor prognosis [1, 2] specially in advanced stages, and a better understanding of the signalling pathways that lead to tumour invasiveness can be fundamental to develop better therapies. The clinical stage and histological grade are the most used classifications criteria defined by the World Health Organization (WHO) to determine treatment and prognosis of OSCC [3]. The clinical-stage considers the association of classification criteria according to the size of primary tumour (T), spread to regional lymph nodes (N), and the presence of distant metastasis (M), the TNM staging system. The histological grade refers to cell differentiation of neoplastic cells, in ascending order; the higher the degree, the cells are less differentiated and more aggressive.

Aggressive tumours have high invasive capacity, which is related to phenotypic alterations between neoplastic cells and surrounding microenvironment. This altered cell behaviour modulates tumour invasion by triggering cell invasion pathways that often result in increased degradation of the extracellular matrix (ECM) [4, 5].

Invadopodia are actin-rich protrusions on the surface of invasive neoplastic cells that promote the degradation of ECM via localised proteolysis and cell projections, which is mediated by matrix metalloproteinases (MMPs) [6, 7]. In tumour cells, these structures contribute to the penetration of the basal lamina and may lead to metastasis [8].

There are some pathways involved in invadopodia formation. The epidermal growth factor receptor (EGFr) signalling pathway is one of the most important initiators for the formation of these finger-like structures and their degradative activity [7]. Proteins related to the regulation of the actin cytoskeleton modulated by EGFr signalling are essentially involved [9]. In addition, the hypoxic microenvironment is also one of the stimuli for invadopodia formation [10]. Following EGFR signalling, the proto-oncogene tyrosine-protein kinase (Src) pathway also appears to initiate invadopodia formation, phosphorylating scaffold proteins such as tyrosine kinase 4 (TKs4), TKs5 and cortactin [11].

Cortactin is a cytoplasmic protein that promotes polymerisation and the rearrangement of the actin cytoskeleton. Thus, it has a fundamental role on the dynamics of the invadopodium [12]. The overexpression of cortactin is observed in different types of cancers and is linked to the process of cell migration, invasion and metastasis [5–12].

In addition, when the Src pathway is activated in cancer cells, the TKs5 substrate is upregulated leading to degradation of the ECM and inducing invadopodia formation; this relationship has been ratified in prostate cancer [13]. TKs5 domains also have the ability to interact with proteins other proteins, such as cortactin and a disintegrin and metalloproteinases (ADAM) family [14, 15]. Ongkeko et al. [16] observed the overexpression of TKs5 in head and neck carcinomas and emphasised that the presence of these markers in cancer are important since they may be the target of therapies that inhibit their activity.

Another important tyrosine kinase in the invadopodia signalling pathway is TKs4. This protein was first described by Buschman et al. [17], who observed its influence on the formation of invadopodium and the degradation activity of the ECM, by regulating the proteolytic activity and recruitment of the metalloproteinase type 1 membrane (MT1-MMP) enzyme. Lányi et al. [18] suggested the interaction of TKs4 with cortactin, as well as TKs5, influences the polymerisation of actin.

MT1-MMP is the major metalloproteinase associated with invadopodia invasion [19]. MT1-MMP is locally recruited and/or stabilised by the activity of TKs4 in the invadopodia front, promoting ECM degradation and focal invasion [20]. In addition, MT1-MMP has the ability to recognise and cleave a variety of cell active substrates and activates MMP2 [21], resulting in higher invasive potential of tumour cells. Thus, this study has the objective of investigate the immunoexpression of the major invadopodia related-proteins TKs5, TKs4, cortactin, and MT1-MMP, correlating their expression with samples of OM, and clinical and histological stage of primary OSCC.

## Methods

This is a cross-sectional, descriptive observational study using tissue microarray (TMA OR601c, US Biomax Inc., Rockville, MD, USA) containing 50 samples of OSCC and 10 samples of healthy oral mucosa (OM) used as control. Ten cases of healthy OM from a histopathology laboratory were added to the control group from tissue TMA, performing a total of 20 samples in control group. The TMA samples comprised of the specimens themselves and clinical data from patients. This study was performed according to the criteria established by the Ethics Committee on Human Research of the Health Sciences Institute of the Federal University of Pará - ICS/UFPA and approved under protocol number 2.976.544.

OSCC were classified according to the 2017 WHO classification of head and neck tumours for histological grades and histological stage [3]. Regarding histological grading, tumours were classified as Grade I (GI), well differentiated (Tissue architecture similar to the normal pattern of squamous epithelium); GII, moderately differentiated (Some degree of pleomorphism and mitotic activity Little keratinization); GIII, poorly differentiated (Predominance of immature cells Abundant typical and atypical mitoses Minimal keratinization). Tumour staging used the standard TNM classification, were stage I (SI) comprised of T1N0M0; stage II, T2N0M0; stage III, any of the following combination T3N0M0, T1N1M0, T2N1M0, T3N1M0. Samples from stages 0 or IV weren´t included in this study [3].

For the immunohistochemical assessment, the slides were deparaffinised in xylol and hydrated in decreasing concentrations of ethanol. They were then immersed in 6 % hydrogen peroxide and methanol in a ratio of 1:1 for 20 min to inhibit the endogenous peroxidase activity. Antigen retrieval was performed in citrate buffer (pH 6.0) in a Pascal pressure chamber (Dako Cytomation®, Carpinteria, CA, USA) for 30 s. Non-specific binding sites were blocked with 1 % bovine serum albumin (Sigma-Aldrich Corp., St. Louis, MI, USA) in a phosphate-saline buffer (PBS) for 1 h. OSCC and OM tissues were incubated with the primary Anti-TKs5 (1:100, Sigma®), Anti-cortactin (1:50, Sigma®), Anti-TKs4 (1:25, Biorbyte, Cambridge, UK) and Anti-MT1-MMP (1:25, R&D Systems, Minneapolis, USA) for 1 h. The slides were incubated for 30 min with the EnVision Plus (Dako®) detection system. Diaminobenzidine (DAB) (Dako®) was used as a chromogen. Posteriorly, the slides were counterstained with Mayer’s haematoxylin (Sigma®) and mounted with Permount (Fisher Scientific, Fair Lawn, NJ, USA).

Five images were taken per case and were saved in the TIFF format. The ImageJ software was used for the semiautomatic image analysis. The colour detection of the DAB staining was performed by the IHC Toolbox plugin, developed by Shu et al. [22], which can be effectively used to analyse immunohistochemically stained samples. The model for the identification of brown pixels was adjusted for the present study. After detecting the DAB colour, the RGB colour images were converted to 8-bit files. Colour inversion was performed, and the mean intensity of the greyscale pixels was obtained.

The data obtained were analysed on the GraphPadPrism 8 software (GraphPad Software Inc., San Diego, CA, USA). The difference between the expression of the proteins in the control group and OSCC was verified by the non-parametric Mann-Whitney U test. The difference between the threegroups was verified by the Kruskal-Wallis test with Dunn’s post-test. A 95 % confidence interval was assumed.

## Results

The clinicopathological data of patients with OSCC are shown in Table 1. In the samples studied, the mean age was 56-years-old with 54 % of individuals equal or below this age and the remaining cases (46 %) were from patients older than it. Males were the most prevalent, with 66 % of the cases. Regarding the histological grade, the cases ranged between grades I to III (GI, GII and GIII) of the histological differentiation with most of them (54 %) corresponding to GI, followed by GII (38 %). In relation to the clinical-stage, 52 % were classified in stage I, 28 % in stage II and 20 % in stage III.

**Table 1:** Clinicopathological characteristics of the 50 cases of OSCC

Variables	Categories	*n* (%)
Age	≤ 56	27 (54)
	> 56	23 (46)
Gender	Male	33 (66)
	Female	17 (34)
Histological grade	I	27 (54)
	II	19 (38)
	III	4 (8)
Clinical stage	I	26 (52)
	II	14 (28)
	III	10 (20)

**Fig. 1:**
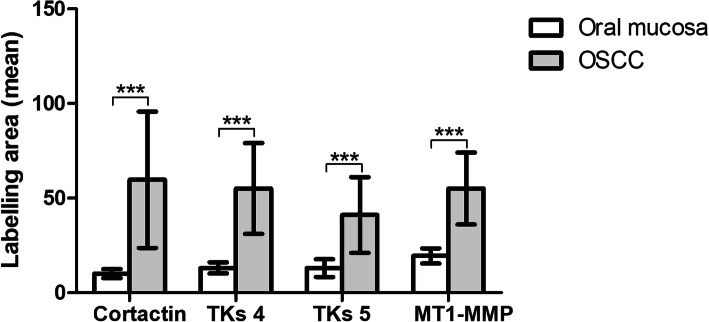
Comparison of the immunoexpression of TKs5, cortactin, TKs4 and MT1-MMP between the samples of OM and OSCC, *** *p* < 0.0001

**Fig. 2:**
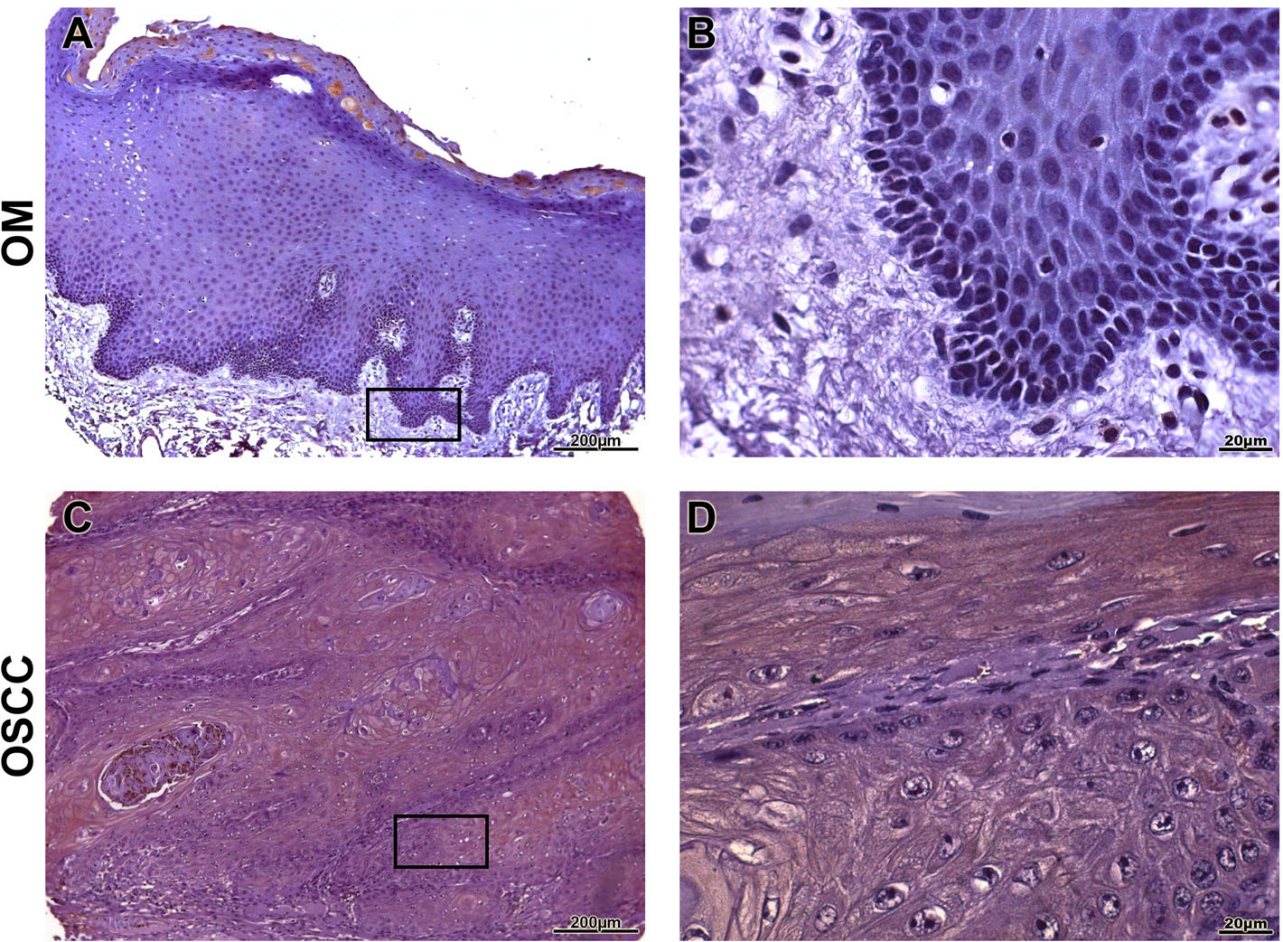
Immunohistochemical analysis of TKs5. Immunoexpression in oral mucosa (**a**, **b**). Cytoplasmic expression of TKs5 in tumour parenchyma cells (**c**, **d**). Immunoperoxidase. Scale: 200 μm (**a**, **c**) and 20 μm (**b**, **d**)

**Fig. 3:**
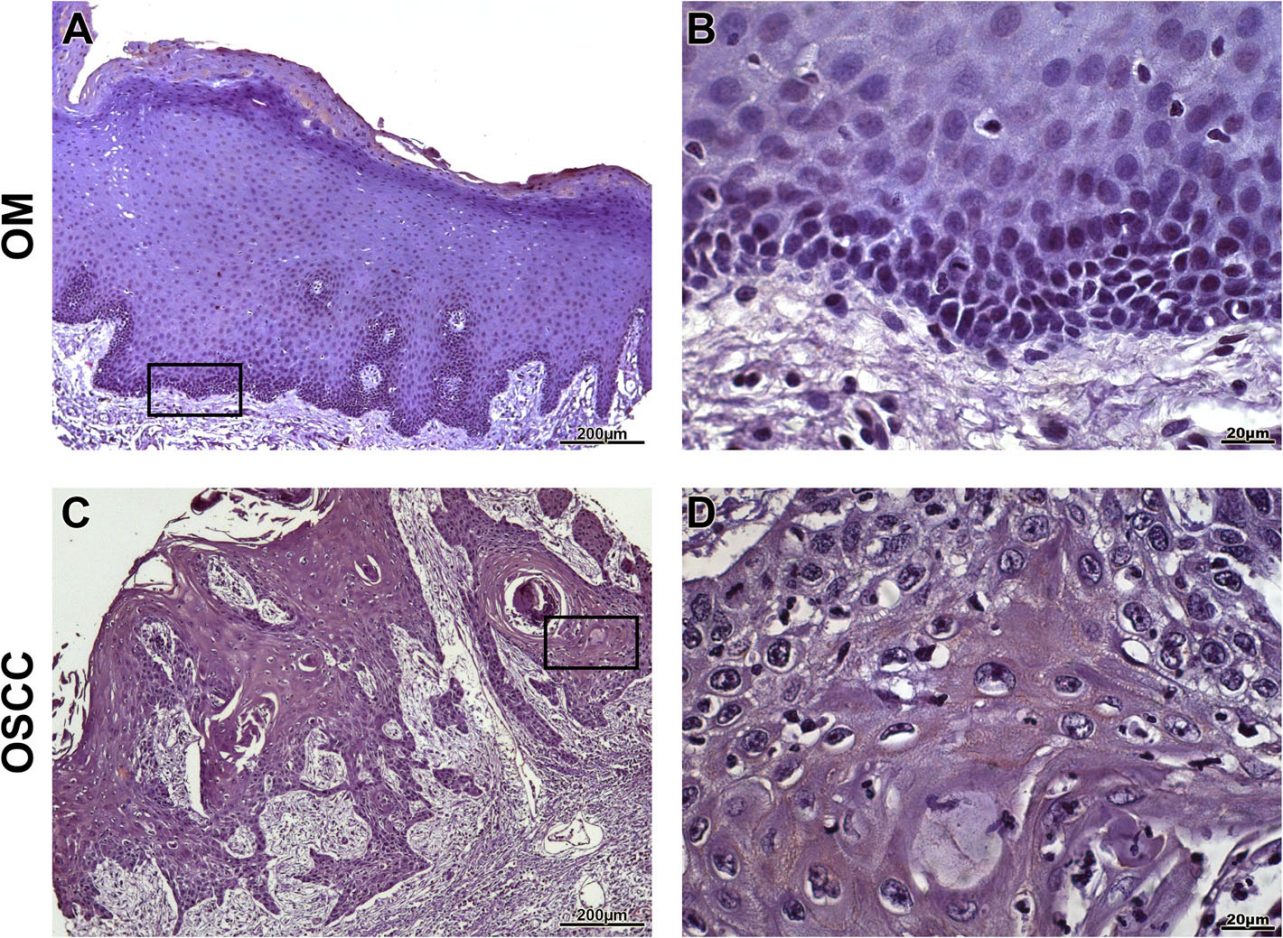
Immunohistochemical analysis of TKs4. Immunoexpression in oral mucosa (**a**, **b**). Cytoplasmic expression in tumour parenchyma cells (**c**, **d**). Immunoperoxidase. Scale: 200 μm (**a**, **c**) and 20 μm (**b**, **d**)

**Fig. 4:**
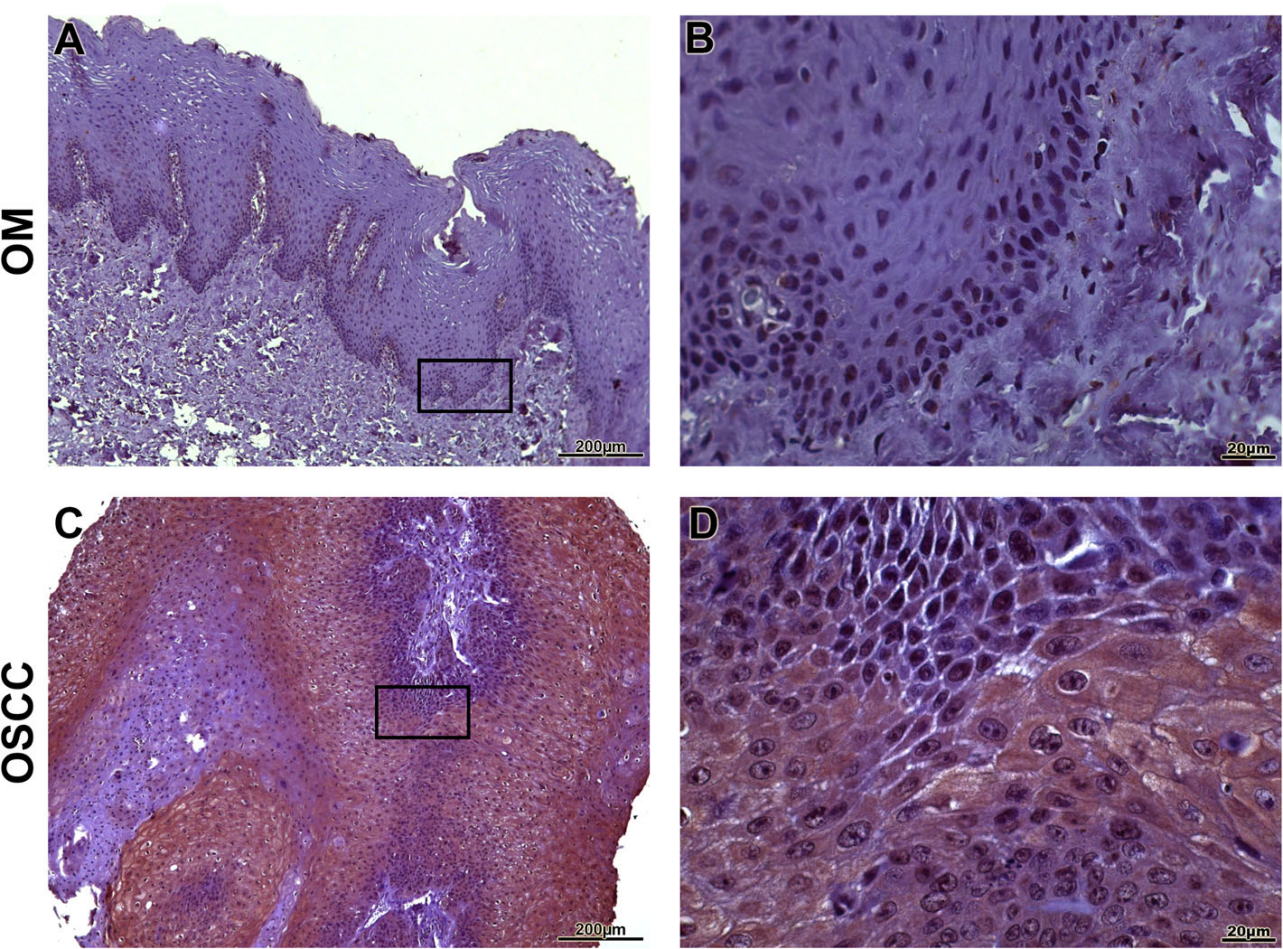
Immunohistochemical analysis of cortactin. Immunoexpression in oral mucosa (**a**, **b**). Cytoplasmic expression in cells of the tumour parenchyma and plasma membrane (**c**, **d**). Immunoperoxidase. Scale: 200 μm (**a**, **c**) and 20 μm (**b**, **d**)

**Fig. 5:**
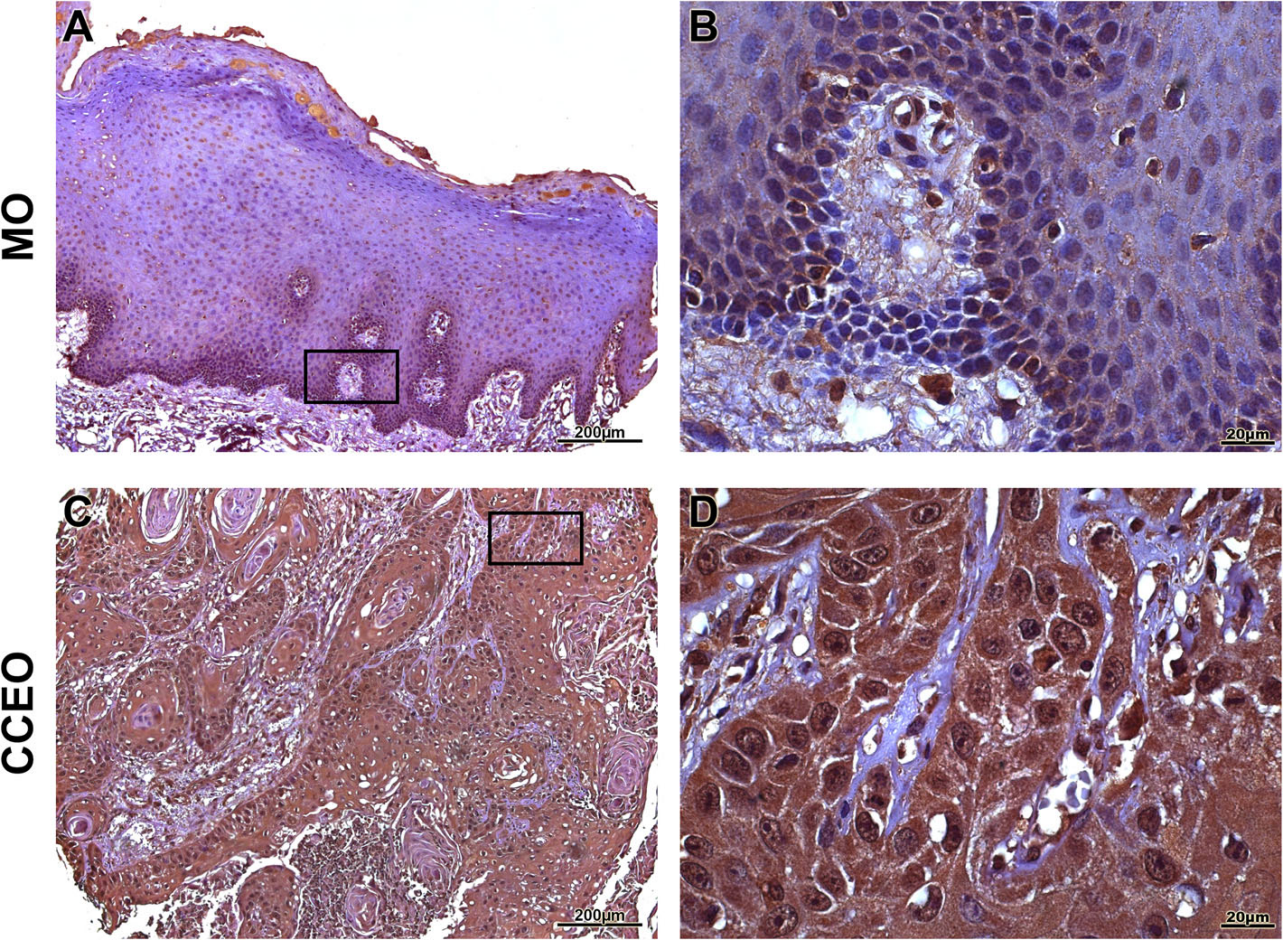
Immunohistochemical analysis of MT1-MMP. Immunoexpression in oral mucosa (**a**, **b**). Cytoplasmic expression in cells of the tumour parenchyma and cell membrane (**c**, **d**). At a lower magnification, the predominant immunostaining of the cells of the periphery of the epithelial cords (C) was observed. Immunoperoxidase. Scale: 200 μm (**a**, **c**) and 20 μm (**b**, **d**)

OSCC samples showed a statistically significant expression of TKs5, cortactin, TKs4 and MT1-MMP when compared to OM controls (*p* < 0.0001) (Fig. 1). The expression of Tks5 (Fig. 2) and TKs4 (Fig. 3) were observed in the cytoplasm of tumour parenchyma cells. Cortactin was expressed in the cytoplasm and cell membrane (Fig. 4). The MT1-MMP was localised in the cytoplasm and cell membrane, predominantly in the peripheral cells of the epithelial cords and islands (Fig. 5).

**Fig. 6:**
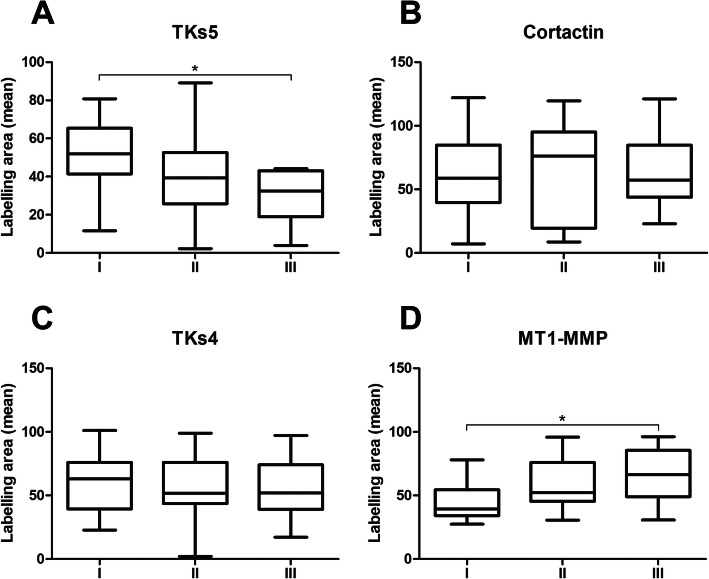
Comparison of the immunoexpression of the key proteins of the invadopodia in the different clinical-stages. Tks5 was more expressed in stage I and presented statistical difference with group II (*p* = 0.026) (**a**). Cortactin and TKs4 maintained similar values of immunostaining at different clinical-stages (**b** and **c**, respectively). MT1-MMP was expressed more in cases of greater lesion progression (*p* = 0.0185) (**d**)

When comparing the immunoexpression of invadopodia related-proteins between the different clinical-stages in OSCC. A decrease in TKs5 expression was noted while tumour progressed from stage I to III. There was a statistically significant difference in the immunolabelling of TKs5 between groups I and III (*p* = 0.026) (Fig. 6 a). The expression of cortactin and TKs4 were relatively stable in the three clinical staging groups with no statistical difference (Fig. 6b and c, respectively). Inversely to TKs5, MT1-MMP showed a tendency to increase its expression as tumour stages progresses, showing a statistical difference between stages I and III (p = 0.0185) (Fig. 6D).

**Fig. 7:**
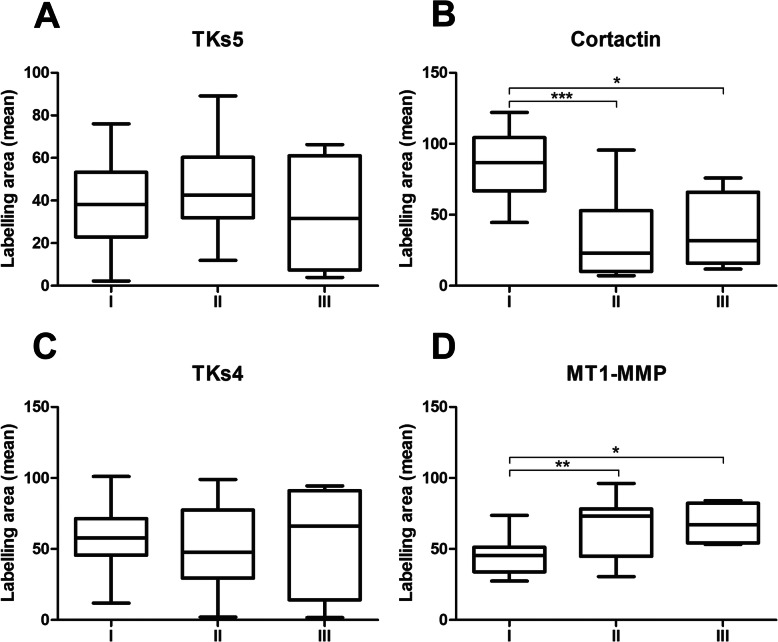
Comparison of immunoexpression of the key proteins of the invadopodia in the different histological grades. Tks5 did not present statistical difference in the different groups (**a**). Cortactin was more expressed in degree I of differentiation in relation to grade II and III (**b**). Immunostaining values for TKs4 did not present statistical differences in the different groups (**c**). MT1-MMP was more expressed in cases of lower cell differentiation (**d**). * *p* < 0.05, ** *p* = 0.001, *** *p* = 0.0001

Regarding the histological grade, Tks4 and Tks5 didn´t show statistical difference among the histological grades (Fig. 7: a and c, respectively). Cortactin immunostaining was statistically higher in GI than in GII and GIII (Fig. 7b). On the other hand, cases with less undifferentiated cells (GI) showed lower MT1-MMP expression when compared to GII and GIII (Fig. 7d).

## Discussion

Invadopodia is one of the major mechanisms that metastatic neoplastic cells with high invasion capacity use the to spread to other tissues [5, 13, 23]. Our results showed overexpression of invadopodia proteins in OSCC in comparison with OM, suggesting the invadopodia is active in OSCC and may be behind of the unfavourable prognosis observed in this tumour [24]. Immunostaining of MT1-MMP, the most important protease involved in ECM in invadopodia front, increased according to tumour progresses to less favourable clinical-stages and histological grade.

The neoplastic progression that triggers metastasis is a complex process that has been attributed, in part to invadopodia, due to their capacity of focal invasion of the ECM [20, 25]. Epithelial cancer cells are subject to several stimuli that can activate the EGFr signalling, resulting in phosphorylation of TKs5. TKs5 then recruits and activates cortactin [26]; therefore, initiating actin rearrangement and polymerisation [5]. Cortactin [27] and Tks4 [17] are related to the recruitment of proteases to invadopodia front; but TKs4 plays a leading role in the localisation and stabilisation of MT1-MMP, resulting in activation of this and other MMPs. Because of this crosstalk between TKs5, cortactin, Tks4 and MT1-MMP, these proteins are recognised as key proteins for invadopodia formation and function.

Stylli et al. [28] verified the participation of TKs5 in the formation of invadopodia as an important component in the promotion of actin projections in melanoma. Another study has reported the expressive presence of TKs5 in breast and colon cancer, especially in cases of worse prognosis [29]. In prostate cancer, high expression of TKs5 was observed in high-grade tumours [13], suggesting that overexpression of TKs5 was associated with higher aggressiveness of this lesion. In our samples, the expression of TKs5 was uniform independent of cell differentiation, suggesting the overexpression of this protein was independent of the histological grade. However, in stage I, TKs5 expression was higher than in stage III, showing a decreasing tendency in advanced tumours.

Increased cortactin expression has been observed in OSCC and associated with spreading to lymph nodes. Eleven of 39 cases where cortactin was overexpressed showed lymph nodes invasion, suggesting this protein as a prognostic marker for invasive and metastatic OSCC [30]. Hofman et al. [31] evaluated 77 cases of OSCC and found a correlation between advanced clinical staging, histological grade and lower survival with higher expression of cortactin. Indeed, cortactin was overexpressed in OSCC compared to OM, however, we found no correlation between clinical stage and a higher expression in well differentiated tumours (GI) than in GII and GIII. These results suggest that more investigations are needed to clarify the role of cortactin as a determinant of prognosis marker. For instance, in oesophageal SCC, higher scores on cortactin immunolabelling were significantly associated with a higher histological grade and a worse prognosis; however, when a multivariate analysis was carried out, higher scores of cortactin weren´t an independent risk factor for lower survival rate [32].

There are few studies that have evaluated the expression of TKs4 in oral lesions. Ribeiro Ribeiro et al. [33] observed the expression of TKs4, cortactin, TKs5 and MT1-MMP in odontogenic keratocyst and found that these proteins were overexpressed in this aggressive odontogenic benign lesion, suggesting the formation and participation of invadopodia in the progression of this lesion. In melanoma, the expression of TKs4 was classified as high in both, metastatic and non-metastatic tumours, with no statistical difference between them [34].

In malignant epithelial tumours, neoplastic invasion has in the basal lamina its first physical barrier to transpose, so the initial steps for tumour dissemination requires localized proteolytic remodelling [25]. The basal lamina is composed mainly of type IV collagen and laminin [35], which protease degradation is a fundamental step for tumour cells to reach the adjacent tissues [36]. MT1-MMP was the first membrane-anchored metalloproteinase to be discovered and currently it stands out for its overexpression in epithelial malignancies [36, 37].

The physiological expression of MT1-MMP on the cell surface is generally low since it is rapidly internalised by endocytosis. However, its increased concentration in the plasma membrane is correlated with malignancy [38]. Elevated levels of MT1-MMP that cause ECM remodelling are associated increased trafficking of proteases in microtubule of regions were invadopodia is formed. The interaction of MT1-MMP-containing endosomes and the actin network are responsible for the recycling and stabilisation of this protein [25].

In this study, we found MT1-MMP overexpressed in OSCC, which is a common finding in malignancies [39]. The role of MT1-MMP has been studied *in vitro* using breast cancer cells and melanoma, which demonstrated that the ECM degradation mediated by invadopodia is MT1-MMP-dependent [40]. MT1-MMP accumulation and activity is observed in regions forming invadopodia in breast cancer cells, which reinforces the participation of this protease in invadopodia physiopathology and tumour progression [25].

Indeed, invadopodia is present not only in established SCC, but its expression is also seen in premalignant nondysplastic and dysplastic lesions. Ali et al. showed that progressing premalignant lesions that evolved to SCC have higher invadopodia scores, which were based on invadopodia markers, than non-progressing lesions [41]. This finding places invadopodia as an important marker for predicting malignant transformation and a potential determinant of treatment, where high-risk cases could require further aggressive treatment.

It is well-known that chronic inflammation creates an environment that helps the development of many cancers. A TNF-driven oral inflammation promotes a pro-inflammatory environment and proinvasion phenotype leading to the recruitment and activation of inflammatory cells and invadopodia formation [42]. An increase in local neutrophil infiltration is seen in aggressive cases with decreased survival rate [42] and also in progressing cases that undergo malignant transformation [41]. The identification of invadopodia promotors and regulators are quite important and can be a target for specific drugs development and anticancer therapy [43].

Thus, our results showed MT1-MMP expression as an important marker in OSCC. MT1-MMP immuoexpression gradually increased according tumour progresses from GI to GIII and from stage I to stage III. While clinical-stage and histological grade are so far the most used parameters to determine prognosis, our results have demonstrated a correlation between MT1-MMP expression and cancer progression, which can be used as a prognostic marker in OSCC.

## Conclusions

To our knowledge, this is the only study showing the combined expression of the invadopodia related-proteins TKs5, cortactin, Tks4 and MT1-MMP in OSCC. These are likely the most important proteins, which play a crosstalk regulating invadopodia activity. These invadopodia related-proteins were overexpressed in OSCC, suggesting invadopodia formation and activity, which can be underlying the focal invasion and poor prognosis seen in OSCC. The development of therapies that target these proteins preventing them to properly form invadopodia can be a promising tool in the treatment of OSCC and other cancers that uses similar invasive mechanisms. While cortactin, TKs4 and TKs5 showed no or ambiguous differences in protein expression when compared between different clinical-stages and histological grades, MT1-MMP immunoexpression increased according tumour progresses to more advanced stages and aggressive grades, suggesting it as a valuable prognostic marker in OSCC.

## Data Availability

The datasets used and/or analysed during the current study are available from the corresponding author on reasonable request.

## Abbreviations

DAB: Diaminobenzidine
ECM: Extracellular matrix
EGFr: Epidermal growth factor receptor
M: Occurrence of metastases
MMPs: Matrix metalloproteinases
MT1-MMP: Metalloproteinase type 1 membrane
N: Involvement of lymph nodes
OM: Oral mucosa
OSCC: Oral squamous cell carcinoma
SRC: Proto-oncogene tyrosine-protein kinase Src
T: Tumor size
TKs4: Tyrosine kinase 4
TKs5: Tyrosine kinase 5
TMA: Tissue microarray
WHO: World Health Organization
